# High ejection fraction of the left ventricular trabecular layer of the human heart

**DOI:** 10.14814/phy2.16101

**Published:** 2024-06-21

**Authors:** Ionela‐Simona Visoiu, Roxana Cristina Rimbas, Alina Ioana Nicula, Dragos Vinereanu, Bjarke Jensen

**Affiliations:** ^1^ Department of Cardiology and Cardiovascular Surgery University of Medicine and Pharmacy Carol Davila Bucharest Romania; ^2^ Department of Cardiology University and Emergency Hospital Bucharest Romania; ^3^ Department of Radiology University and Emergency Hospital Bucharest Romania; ^4^ Department of Medical Biology, Amsterdam Cardiovascular Sciences University of Amsterdam, Amsterdam UMC Amsterdam the Netherlands

**Keywords:** cardiomyopathy, heart failure, non‐compaction

## Abstract

Numerous diagnostic criteria for excessive trabeculation, or “noncompaction,” score the extent of the trabecular layer. Whether the trabeculations themselves have a poor or good contractility is largely unknown. We retrospectively analyzed cardiac magnetic resonance (CMR) of patients with excessive trabeculation of the left ventricle (LV). The LV was labeled into four regions: compact wall, central cavity (CC), trabeculations, and intertrabecular recesses (IR). For each label we calculated the systolic fractional volume change (SFVC) in short‐axis images (*n* = 15) and systolic fractional area change (SFAC) in four‐chamber images (*n* = 30). We measured the ejection fraction (EF) of IR, CC, and total cavity. Three methods to calculate EF of the total cavity were compared: trabeculations included (per guidelines), IR excluded (Jacquier criterion), and trabeculations contoured and excluded (contour‐EF). The SFVC and SFAC of the compact wall were similar with SFVC and SFAC of trabeculations. In contrast, the IR were more diminished in systole by comparison with the CC, having lower SFVC (39% vs. 56%) and SFAC (37% vs. 72%). EF of the IR was also greater than EF of the CC (61% vs. 44%). Excluding IR from the total cavity or including trabeculations negatively impacts the EF (44% and 40%, respectively, vs. 51% for contour‐EF). The trabecular layer operates at a high EF.

## Graphical abstract

The trabecular layer of the human left ventricle operates with a comparatively high ejection fraction. If this is not accounted for, the actual ejection fraction, which is a key prognostic indicator, is underestimated.

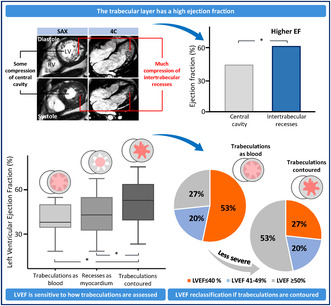

## 1 INTRODUCTION

Key prognostic indicators of heart function, such as left ventricle (LV) cardiac output, stroke volume (SV), ejection fraction (EF), and end diastolic volume (EDV) are currently measured by echocardiography or cardiac magnetic resonance imaging (CMR), both clinically and experimentally (Dewey et al., 2020). In human clinical setting, where most data exist, subtle variations in LV structure and function are now correlated to differences in quality of life and incidence of major adverse outcomes, as suggested by the big data analyses (de la Chica et al., 2020; Meyer et al., 2020; Sigvardsen et al., 2021; Woodbridge et al., 2019). One concern, however, is whether the functional readouts, which are often key prognostic indicators, are measured with the same accuracy across the various ventricular anatomies that the clinician encounters.

**FIGURE 1:**
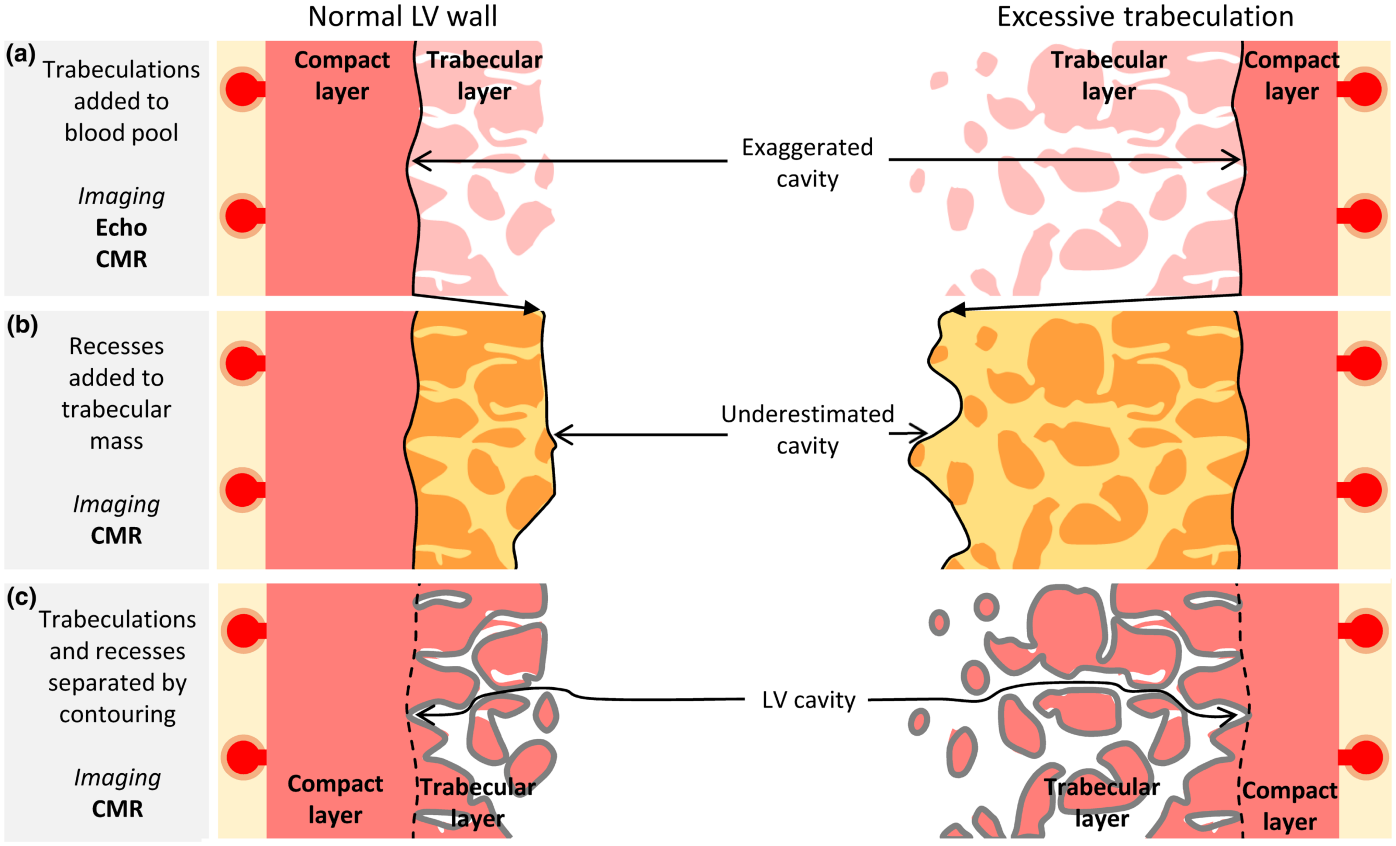
Schematic illustrations of biases in assessing LV volumes. (a) Per guidelines, the trabeculations can be included in the cavity which then exaggerates the LV blood volume. (b) Some quantifications of the trabecular mass (orange area) include the intertrabecular recesses, whereby the trabecular mass is exaggerated and LV blood volume is underestimated. (c) Contouring (gray lines) separates blood and trabeculations, but its accuracy has no independent validation. Error in contouring, which is effectively unavoidable, is illustrated by imperfect lining of the trabecular contours.

Imaging biases are consciously accepted in many instances even if the impact of them is only partly understood (Figure 1). The highly complex boundary between LV blood and trabeculations, for example, cannot be fully recognized with the spatial resolution of conventional imaging (Polacin et al., 2022; Riekerk et al., 2022). In echocardiography guidelines, papillary muscles and trabeculations are deliberately added to the LV blood pool (Lang et al., 2015), and this is also the case in some CMR studies (Davies et al., 2022). While this is a pragmatic approach that strives to achieve highly reproducible assessments, it introduces a bias that affects EF, but not SV, because the unejectable trabecular tissue is measured as blood (Figure 1a). A different bias is introduced when the intertrabecular recesses (IR), which hold LV cavity blood, are added to the myocardial mass (Figure 1b) (Choi et al., 2016; Dreisbach et al., 2020; Jacquier et al., 2010; Stacey et al., 2013). Attempts to avoid such biases have been made by contouring the trabeculations to the ventricular mass and the IR to the LV blood pool (Figure 1c) (Grothoff et al., 2012; Jaspers et al., 2013; Luu et al., 2022; Positano et al., 2018). These methods are in effect the application of a threshold value that is intermediate to the signal intensities of the compact wall and the blood of the central cavity (CC). To what extent such thresholding is accurate is difficult to establish (Thut et al., 2023), because the thresholding is often performed on the images that are themselves considered the golden standard of cardiac imaging. A method of independent validation of the CMR‐based contouring of the trabeculations is not commonly applied.

A recent meta‐analysis not only reaffirmed that when trabeculations are analyzed as being part of the ventricular cavity the EF is lower (worse) and end diastolic and end systolic volume readouts are inflated (worse). It further showed that these biases are greater in a setting of hypertrophy (Zhan et al., 2024). Together with presence of fibrosis, lowered EF and increased EDV are key prognostic indicators in heart failure (McDonagh et al., 2021). The extent of trabeculation is extreme in individuals with excessive trabeculation, also known as noncompaction, or hypertrabeculation, or persistent sinusoids (Petersen et al., 2023). Yet the functional assessment of such individuals typically follow the guidelines that were developed for LVs with a normal extent of trabeculation (Figure 1) (Lang et al., 2015). How much biases in image analyses affect the assessment of such individuals is poorly understood, but the functional interpretation of the anatomical setting of excessive trabeculation is ambiguous as individuals can present with poor pump function while many are asymptomatic (Petersen et al., 2023; Ross et al., 2020; Zemrak et al., 2014). There is then a need to better understand how the trabecular layer contributes to cardiac function. Whether the trabeculations themselves have a poor or good contractility is largely unknown. This study aimed to clarify this issue by measuring the EF of the trabecular layer. It is remarkable that some animals with highly trabeculated ventricles and a very thin compact wall can achieve an EF of 90% (Williams et al., 2019). We hypothesized that the trabecular layer operates at higher EF than the CC. To test the hypothesis, we measure for the first time the EF of the trabecular layer and the CC. This proof of concept study focuses on individuals with excessive trabeculation because, first, their trabecular layer is larger than normal and the EF will be correspondingly easier to measure, and second, the functional assessment of such individuals in particular may be affected by biases in image analyses and may therefore be in greater need of correction. We find consistently that the trabecular layer has a higher EF than the CC across a broad spectrum of LV function, from severe heart failure to normal pump function.

## 2 METHODS

### 2.1 Study design and population

We conducted a retrospective study on patients evaluated by CMR and diagnosed with excessive trabeculation in one center (University and Emergency Hospital, Bucharest, Romania). The inclusion criteria were positive Jacquier criterion (Jacquier et al., 2010) (the trabecular myocardium comprises more than 20% of the LV mass), presence of sinus rhythm, and age over 18 years. We excluded patients with chronic coronary syndromes, significant valvular heart diseases, pericardial diseases, or poor quality of CMR images. Fifteen patients were included in the study (Table 1). The study was approved by the Research Ethics Committee of the University and Emergency Hospital (Bucharest, Romania) (approval number: 74155), and complied with the Declaration of Helsinki. All participants provided informed written consent.

**TABLE 1:** Characteristics of the study population.

	ET patients (*n* = 15)
Age (years)	48.3 ± 12.5
Male (%)	53.3
BMI (kg/m^2^)	29.5 ± 5.9
Systolic blood pressure (mmHg)	135.9 ± 25.5
Diastolic blood pressure (mmHg)	81.7 ± 14.4
Trabeculations % of total LV myocardium	36.3 ± 8.3
Trabeculations volume (mL)	37.0 (33.5–70.5)
Trabecular layer volume (mL)	130.0 ± 48.9
Trabeculations % of the trabecular layer	37.3 ± 7.4

*Note*: Continuous variables are presented as mean ± SD or median (IQR).

Abbreviations: BMI, body mass index; LV, left ventricle.

### 2.2 Cardiac magnetic resonance

CMR was performed using a 1.5‐T MR scanner (Magnetom Sempra, Siemens Healthcare GmnH, Erlanger, Germany). Standard short‐ and long‐axis cine images were acquired using steady‐state free precession sequences. Frames of end‐diastole (ED) and end‐systole (ES) were exported to and analyzed in the 3D software Amira (version 3D 2021.2, FEI SAS, Thermo Fisher Scientific), where all structures were labeled in the Segmentation Editor module. The volumes of labeled structures were retrieved using the Materials Statistics module. To differentiate myocardium from lumen, a mask/signal threshold was applied (per heart) to the ED four‐chamber frame (long axis) or a mid‐ventricular frame (short‐axis stack) such that the papillary muscles were myocardial and the trabecular layer became approximately equal parts trabeculations and IR. Then the threshold was locked and applied to all other ED and ES frames of the images series of that heart. Thus, the LV was labeled into four regions in both ED and ES: compact wall, CC, trabeculations, and IR.

Volumes were measured on the basis of the short‐axis stacks, while areas on the basis of four‐chamber images. For each label, we calculated the systolic fractional volume change (SFVC) and systolic fractional area change (SFAC), by dividing ES to ED values. The volumes of trabeculations and compact wall were calculated as the average of the volumes measured in ED and ES. From the labels of CC and IR we calculated EDV, end‐systolic volume (ESV), SV, and EF. This was done in the three different ways illustrated in Figure 1. In the first way, according to the current guidelines (Lang et al., 2015), the total LV cavity also comprised trabecular myocardium besides the CC and the IR (Figure 1a). In the second way, according to Jacquier et al. (2010), the total LV cavity excluded the IR, which were considered trabecular myocardium (Figure 1b). In the third way, or “contour,” which is the reference in this study, the IR were considered part of the total LV cavity (Figure 1c).

Using syngo.MR Cardiology VB20A post‐processing software (syngo.via, Siemens Healthcare GmnH, Erlanger, Germany), we measured mitral annular plane systolic excursion (MAPSE) and LV length (Rangarajan et al., 2016). MAPSE was calculated as the difference between ED to ES of the wall length, measured as a straight line from epicardial apex to the mitral annulus. We measured MAPSE for all six walls from two‐chamber, three‐chamber, and four‐chamber cine images. We calculated the global MAPSE as the average of all six measurements. LV length was defined as the distance from the epicardial apex and the midpoint of the line connecting the origins of the mitral leaflets. LV length was measured in ED and ES in all three standard apical views. The global LV length was calculated as the average of the three measurements. Based on LV length we calculated the global longitudinal shortening (GL‐shortening), according to the bellow formula, as previously reported (Riffel et al., 2015): GL‐shortening = 100 × (LV_length ED − LV_length ES)/LV length ED.

### 2.3 Analysis of published images

To test the robustness of our findings, we included four‐chamber views of publicly available cases (Barskiy, 2023a, 2023b; Kaplan‐List, 2023; Keshavamurthy, 2023; Luijkx, 2023a, 2023b; O'Rourke, 2023; Sheehy, 2023; Tigges, 2023; Yarmola, 2023) of excessive or abnormal trabeculation from https://radiopaedia.org and from publications (Chan et al., 2019; Martins et al., 2014; Paluszkiewicz et al., 2022; Petersen et al., 2023; Yousef et al., 2009). The inclusion criterion was available images of both ED and ES.

### 2.4 Theoretical model

We used simple theoretical calculations to evaluate the effect of trabeculations on the ventricular performance, using the baseline values listed in Table 2. A normal LV was compared to one with excessive trabeculation, and in both cases the total LV cavity and trabeculations amounted to 150 mL. With the total volume clamped at 150 mL, we then calculated what happened to blood volumes if one parameter such as the EF of the IR varied from 10% to 90%.

**TABLE 2:** Baseline values for theoretical calculations.

	Normal LV			Excessive trabeculation		
	ED (mL)	ES (mL)	EF (%)	ED (mL)	ES (mL)	EF (%)
Papillary muscles	5	5		5	5	
Central cavity	120	48	60	70	28	60
Trabeculations	10	10		30	30	
Recesses	15	6	60	45	18	60

Abbreviations: ED, end‐diastole; EF, ejection fraction; ES, end‐systole; LV, left ventricle.

### 2.5 Statistical analysis

Statistical analysis was performed using the SPSS version 21.0 (IBM Corp., Armonk, NY, USA). Categorical variables were expressed as percentages. Continuous variables were assessed for normality using the Shapiro–Wilk and Kolmogorov–Smirnov tests. Normally distributed continuous variables were reported as mean ± standard deviation (SD), and were compared for statistical significance using one‐sample and paired‐samples *t*‐tests. Non‐normally distributed continuous variables were reported as median and interquartile range (IQR). Correlations between normally distributed continuous variables were performed using Pearson's correlation coefficient. A *p*‐value <0.05 was considered statistically significant.

## 3 RESULTS

### 3.1 Assessment of the trabecular layer based on short‐axis views

**FIGURE 2:**
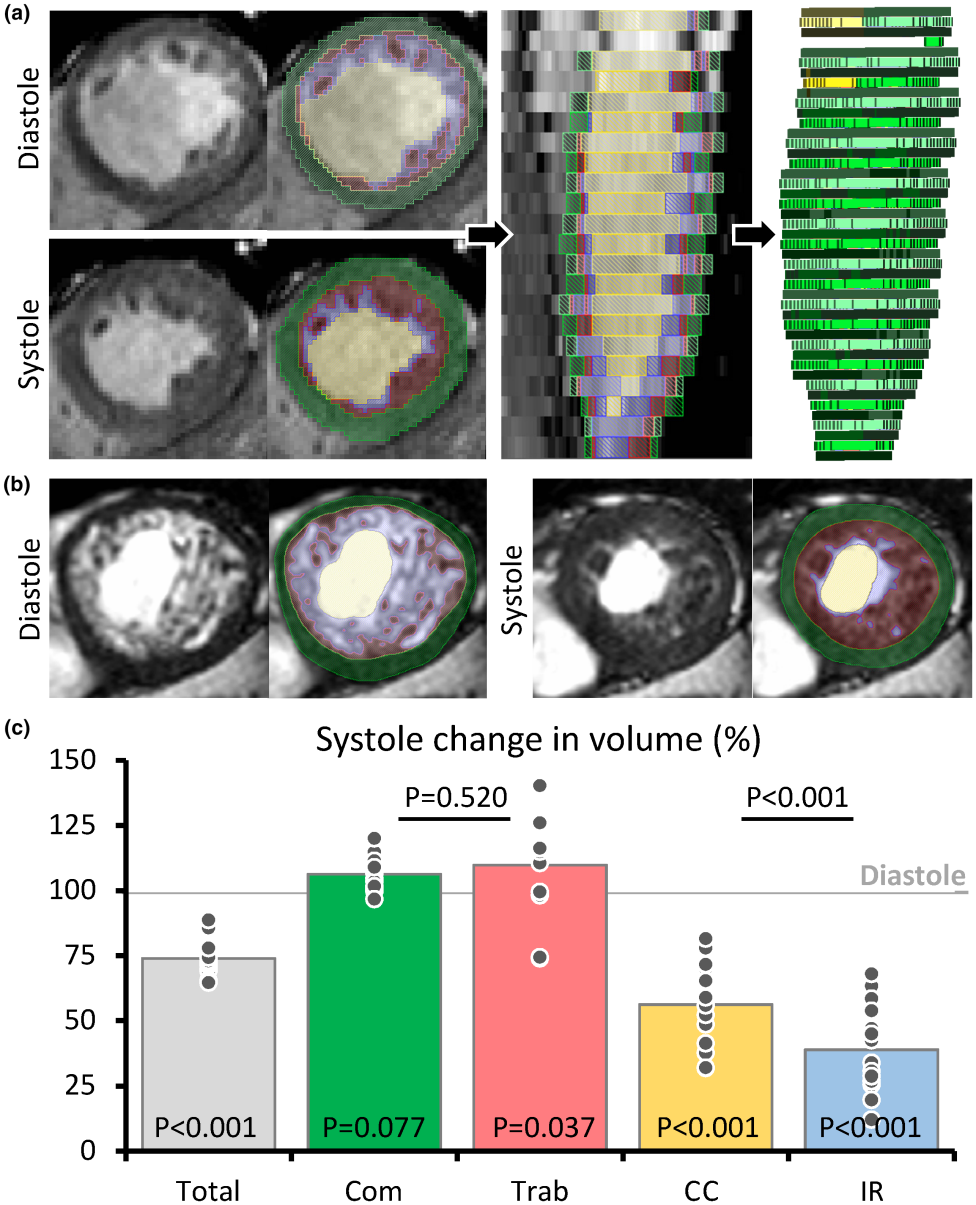
Volume changes of the four major components of the total left ventricle. (a) Example of threshold‐based labelling of a short‐axis slice. (b) Example of threshold‐based labelling of a short‐axis slice from the individual with the greatest proportion of LV trabecular myocardium. (c) Volume change between diastole and systole for the sum of all labels (Total) and each of the four labels (Com, compact layer; Trab, trabeculations; CC, central cavity; IR, intertrabecular recesses). The *p*‐value within each column is the one‐sample *t*‐test for difference from 1 (diastole). The *p*‐values between columns are paired‐sample *t*‐tests for the same mean (*N* = 15).

First, we describe how the volume of the four left ventricular labels change from diastole to systole to document if the readouts of the labelling correspond to expected outcomes (Figure 2a,b). For each label, we measured the SFVC. Total left ventricular volume (tissue and blood) should be smaller in systole, and this was the case (Figure 2c). The volume of myocardium should not be different between diastole and systole, and this was the case for the compact layer, whereas the volume of trabeculations was slightly and significantly greater in systole (Figure 2c). The blood volume should be lower in systole and both the volume of the CC and the IR were substantially lower in systole (Figure 2c). Then, we found that the volume of IR was significantly more reduced by comparison with the CC (Figure 2c) having lower SFVC values (39 ± 17 vs. 56 ± 16%, *p* < 0.001). When the same volume changes were calculated as EF (EDV‐ESV/EDV), the EF of IR was significantly greater than that of the CC (61 ± 17 vs. 44 ± 16%, *p* < 0.001). It required very substantial mislabeling of the CC (deflation) and the recesses (inflation) to make the EF of IRas low as the EF of the CC (Data S1). To further validate the labelling, we compared contour‐EF of the total ventricle to GL‐shortening and MAPSE, the measurements of which can be made without consideration to the trabecular layer. The contour‐EF was positively correlated to both GL‐shortening (*R* ^2^ = 0.341, *p* = 0.021) and MAPSE (*R* ^2^ = 0.268, *p* = 0.045) (Data S1).

### 3.2 Assessment of the trabecular layer based on four‐chamber views

**FIGURE 3:**
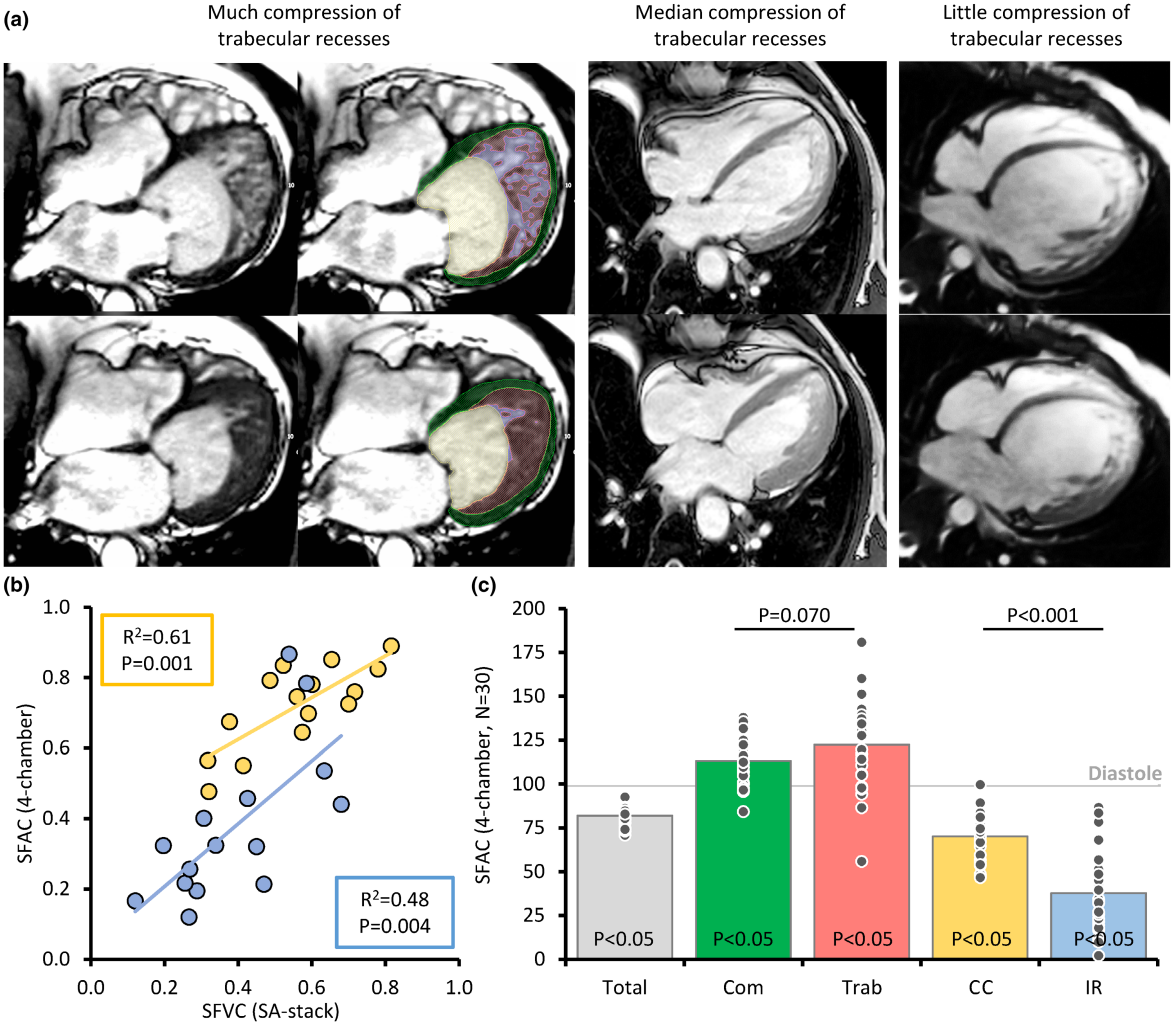
Area changes of the four major components of the left ventricle. (a) Examples of left ventricles in diastole (top row) and systole (bottom row), in which the systolic IR are much compressed (left‐hand image—case from Petersen et al. (2023)), intermediately compressed (middle image—case from Radiopaedia, Luikjx (2023a)), or show little compression (right‐hand image—case from Radiopaedia, Yarmola (2023)). (b) There were significant linear correlations between area (SFAC) and volume (SFVC) changes, both for the CC (yellow) and the IR (blue). (c) Area change between diastole and systole for the sum of all labels (Total) and each of the four labels (Com, compact layer; Trab, trabeculations; CC, central cavity; IR, intertrabecular recesses). The p‐value within each column is the one‐sample *t*‐test for difference from 1 (diastole). Both the compact and trabecular layer areas are significantly greater in systole than in diastole, but not different from each other. In contrast, the intertrabecular recess areas are more diminished in systole than in the CC.

As LV morphology and function are often visualized in four‐chamber view, we made the same analysis for the areas of the four labels in single slice mid four‐chamber view (Figure 3a). First, we correlated the already measured SFVC of the CC and IR from the short‐axis stacks, to the corresponding SFAC from four‐chamber views; area changes were positively correlated to volume changes for both labels (Figure 3b). Given this correlation, we expanded our dataset from 15 to 30 cases using publicly available cases. In systole, the total left ventricular area (tissue and blood) is likely to be smaller, and this was the case (Figure 3c). The area of myocardium is expected to be greater in systole, because the total myocardial volume will be distributed on fewer frames, and both the compact and trabecular layer labels had a greater area in systole (Figure 3c). The cavity areas should be lower in systole, if there is intermediate to good contraction, and this was the case, both for the CC and the IR (Figure 3c). In correspondence to what was found with the volume measurements, the area reduction was significantly greater for IR than for the CC (Figure 3c), having lower SFAC values (37 ± 22% vs. 72 ± 12%, *p* < 0.001, paired *t*‐test).

### 3.3 Impact of the trabecular layer on LV cavity volume assessments

**FIGURE 4:**
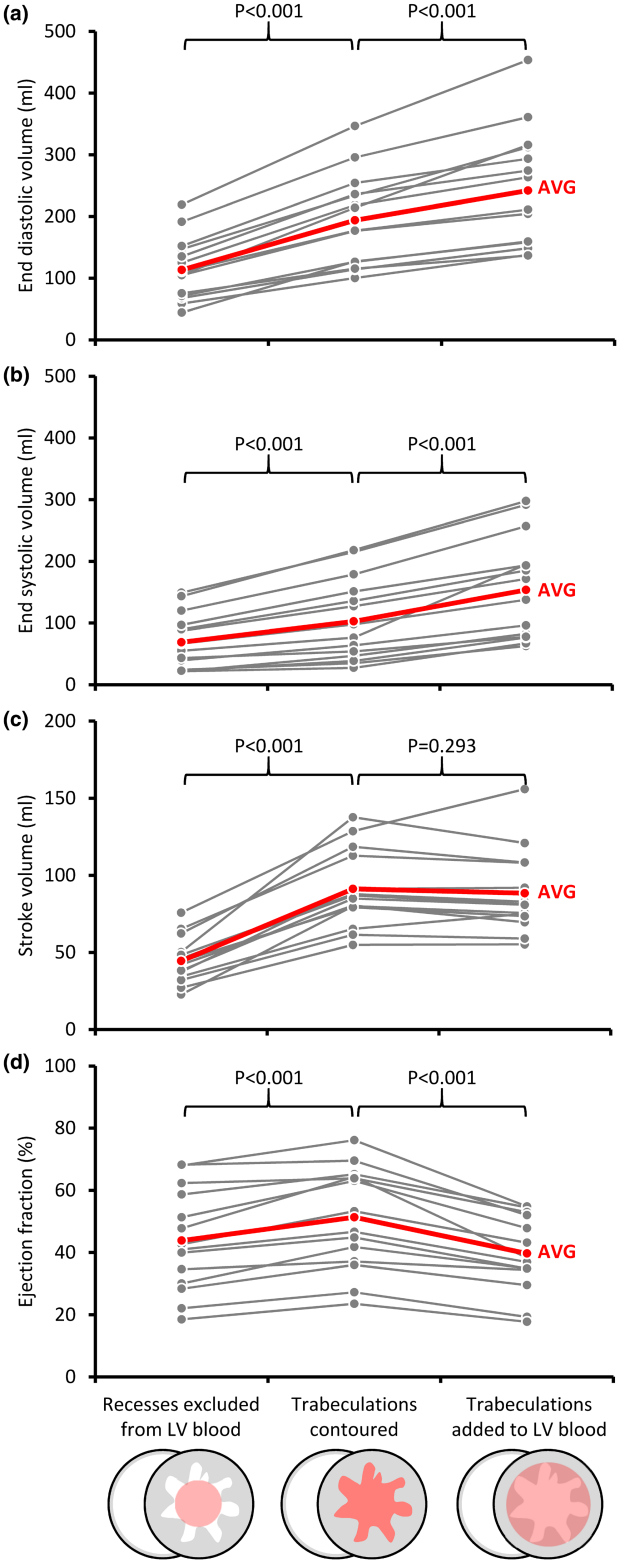
How differences in analyses of the trabecular layer impact on LV cavity volume assessments. (a, b) Compared to trabeculations contouring, the end diastolic (a) and systolic (b) volumes are diminished if recesses are excluded from the blood pool (Jacquier criterion) and are exaggerated if trabeculations are added to the blood pool (per guidelines). (c) The exclusion of the recesses has a negative impact on the stroke volume. (d) The ejection fraction has the highest value if trabeculations are contoured.

Given the higher EF of the trabecular layer than the CC, we next expanded the analysis of our volumetric data to assess the impact of adding the volume of trabeculations to the blood pool, that is the combined volume of the CC and IR (per guidelines). Compared to the volumes derived from contoured trabeculations, EDV and ESV were increased (Figure 4). The SV was not different between the two methods, but given the increased EDV, EF was diminished (Figure 4). We then assessed the impact of excluding the IR from the blood pool per Jacquier criterion (Jacquier et al., 2010). This was done by subtracting the IR volume from LV cavity volume, and then we compared these volumes to the volumes derived from contoured trabeculations. All volumes and the EF were diminished (Figure 4).

### 3.4 Calculations to show how trabecular layer characteristics can affect assessment of volumes

**FIGURE 5:**
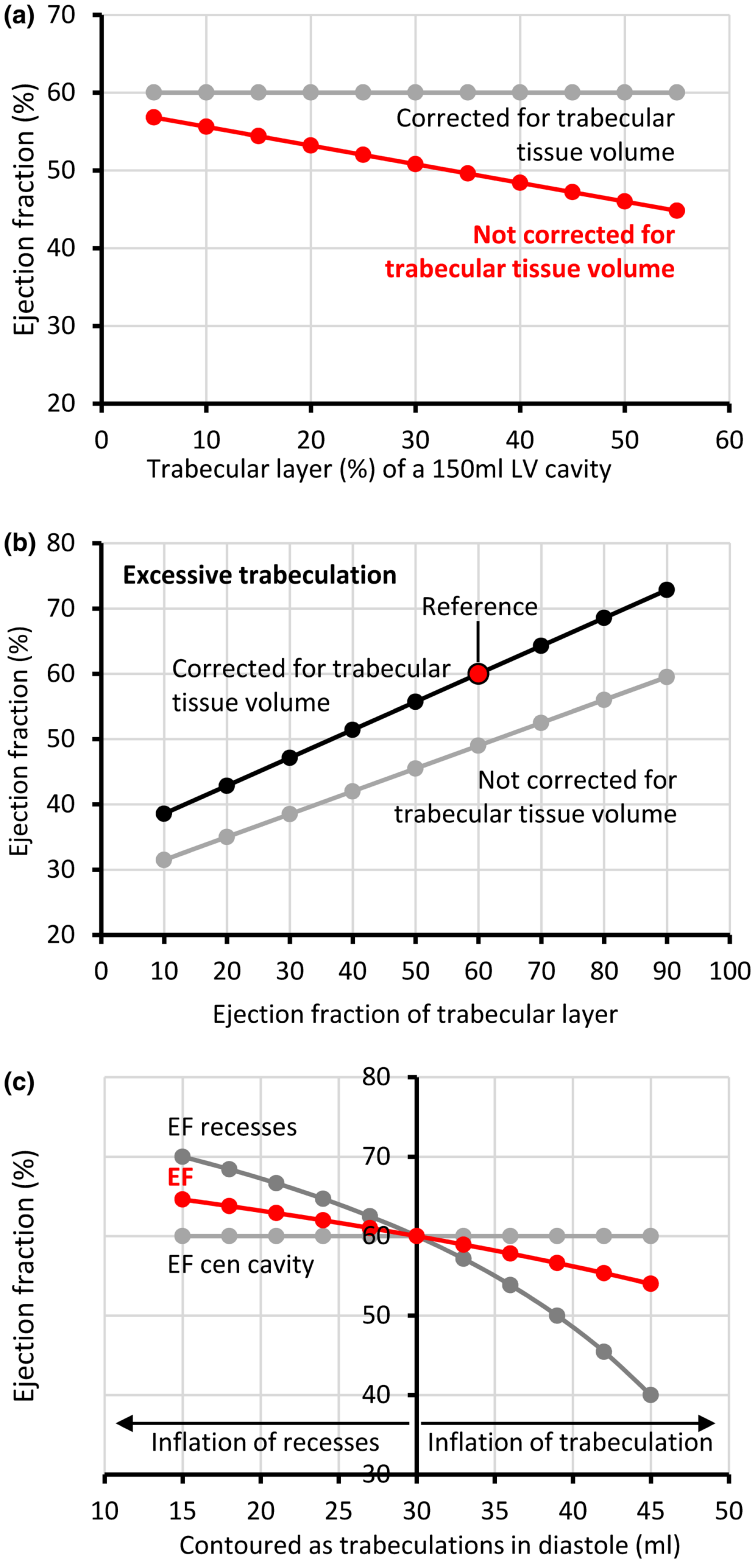
Theoretical permutations to key characteristics of the trabecular layer and its impact on ejection fraction (EF). (a) The greater the proportion of trabecular muscle, the lower the EF, unless the trabecular myocardial volume is corrected for. (b) The greater the EF of the trabecular layer, the greater the total ventricular EF. The EF of the central cavity was kept at 60%. (c) The greater the diastolic measurement of trabecular myocardium deviates from the systolic measurement (30 mL), the greater the perturbation to the EF of the intertrabecular recesses and the total LV EF.

The above analyses suggest the specifics of trabecular layer characteristics, such as EF, can affect volume assessments. Next, we made theoretical permutations to key characteristics, the baseline values of which are listed in Table 2, to assess the scope of these effects. If trabeculations are included in the LV blood pool and the IR and the CC both operate at an EF of 60%, the greater the trabecular tissue volume, the lower the measured EF will be (Figure 5a, Data S1). In failing hearts, the ventricular wall can have much subendocardial fibroelastosis and this can reduce the compliance of the trabecular layer (O'Rourke, 2023; Reyes et al., 2022; Takamatsu et al., 2020). Varying the EF of an excessive trabecular layer from 10% (extremely reduced compliance) to 90%, while keeping the CC EF at 60%, shows the total EF to increase from below 40% to above 70% (Figure 5b, Data S1). Myocardial volume is considered easier to measure in systole (Grothoff et al., 2012) and our own data show the volume measurements of the trabeculations can vary between diastole and systole. If the trabecular tissue volume is underestimated in diastole, it will lead to an overestimation of the EDV, SV, and EF (Figure 5c, Data S1).

### 3.5 LVEF category reclassification after contouring of trabeculations

**FIGURE 6:**
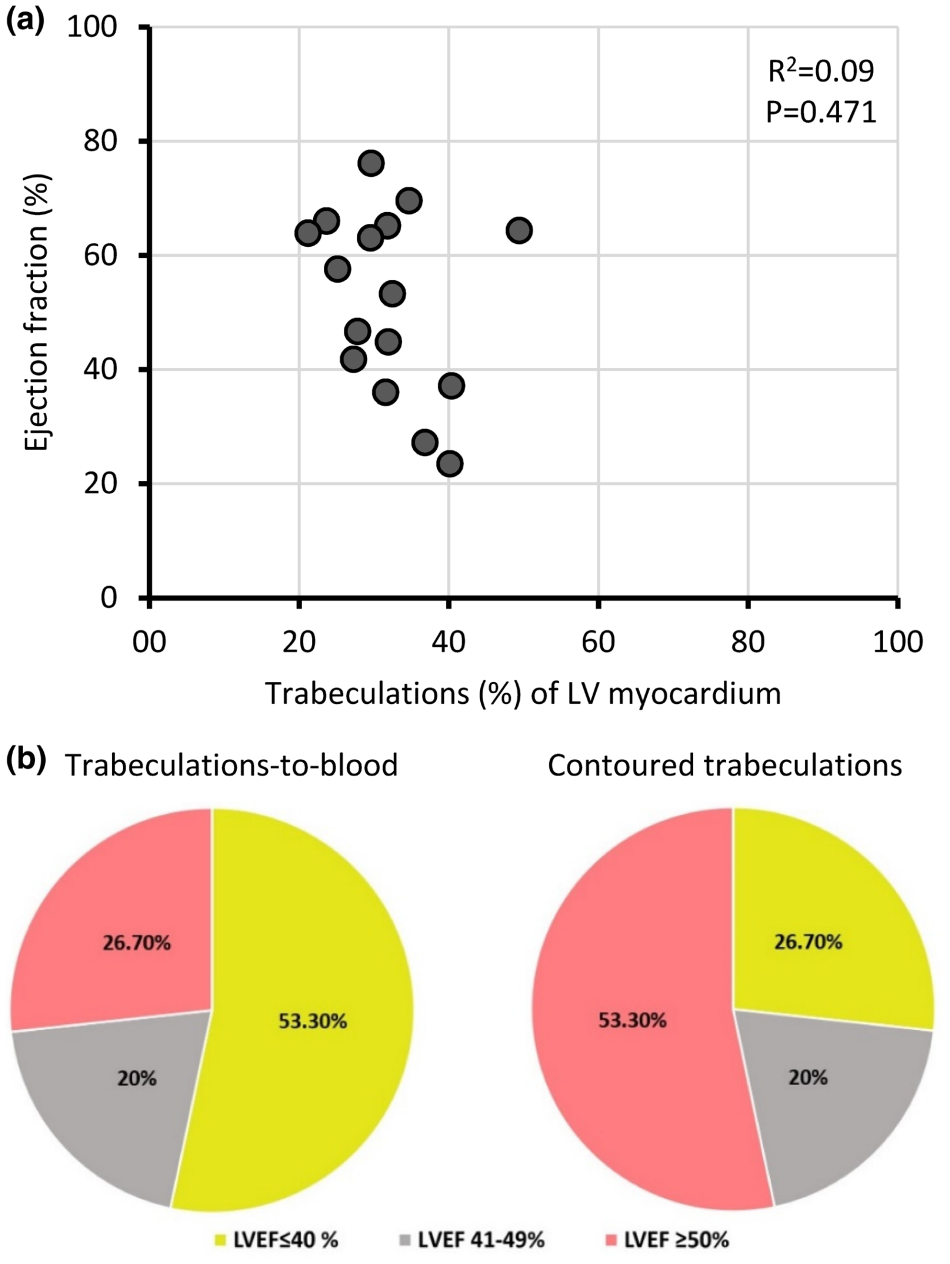
Impact of trabeculations on ejection fractions (EF). (a) When trabeculations were contoured, there was no significant correlation between total left ventricular ejection fraction (LVEF) and the proportion of trabeculation. (b) LVEF category reclassification after contouring of trabeculations.

In the analyses performed so far, there was nothing to indicate that the trabecular proportion of total LV myocardium was predictive of the trabecular layer function. Next, we divided the patient population on the contour‐EF of the total cavity being above or below 50%, and in both of the resultant groups, the EF of the recesses remained significantly greater than that of the CC (Table 3). Also, there was no significant linear correlation between total EF and the extent of trabeculation (*p* = 0.471, Figure 6a). Finally, we classified patients based on contoured trabeculations and per guidelines, and the number of patients with severely reduced EF was halved if the EF was based on contouring of the trabeculations (Figure 6b).

**TABLE 3:** Comparison between the central cavity EF and recesses EF.

Variable	All patients (*n* = 15)	LVEF < 50% (*n* = 7)	LVEF > 50% (*n* = 8)
Central cavity EF (%)	43.8 ± 15.8	30.6 ± 8.7	55.4 ± 10.2
Recesses EF (%)	61.0 ± 16.7	45.9 ± 9.6	74.3 ± 6.9
*p* value	<0.001	0.001	0.002

Abbreviations: EF, ejection fraction; LVEF, left ventricular ejection fraction.

## 4 DISCUSSION

To the best of our knowledge, this is the first study to analyze separately the IR and the CC for the volume changes that occur between diastole and systole. The main finding is that the IR operate at a high EF. This finding is consistent with the reproducible correlation between high levels of physical activity and a greater extent of the trabecular layer (de la Chica et al., 2020; Woodbridge et al., 2019). Conversely, it undermines the notion that the trabecular layer has a direct negative impact on pump function. In the context of so‐called noncompaction cardiomyopathy this notion is widespread, either explicitly or implicitly, but it is a notion that is becoming increasingly untenable (Petersen et al., 2023). Part of the evidence against the notion is that the human ventricular trabecular and compact myocardium is not different in the density of sarcomeres, mitochondria, and vasculature (Faber et al., 2022), and single cell sequencing does not reveal overt differences between trabecular and compact myocardium (Litviňuková et al., 2020). In our interpretation of the current literature, the trabecular and compact layers are made up of similar myocardium. Interestingly, the trabecular and compact layers are transcriptionally the same in tuna fish which has a ventricle that is much more trabeculated than in mammals while the ventricular afterload is mammal‐like (Ciezarek et al., 2020). One difference between the trabecular and compact layers may be that the trabecular layer more readily can collapse on itself than the compact wall, that is, it can achieve a very high EF.

We show that when ventricular volumes and EF are measured, there is a substantial impact of adding the trabeculations to the LV blood pool, as per guidelines (Lang et al., 2015), or removing the IR from the LV cavity, as per a criterion for noncompaction (Jacquier et al., 2010). Only the stroke volume is unaffected, as least as long as the trabecular mass that is added to the LV blood pool, is measured to be the same in both EDV and ESV. The practice of adding trabeculations to the LV blood pool, or removing the IR, likely reflects pragmatic choices to create easily standardized methodologies, even if these practices introduce biases. The challenge is that it is difficult for the clinician to compute on‐the‐spot what these biases are, and derive how they affect key parameters. And these biases are exaggerated if the trabecular layer is greater than normal. The method of adding trabeculations to the LV blood pool is ubiquitous in echocardiography where trabeculations cannot yet be reliably contoured. Perhaps the simplest approach to deal with the resultant bias is to consider the readouts of EDV, ESV, and EF as likely to have more error the greater the trabecular layer. To the extent that important diagnostic choices are made on the basis of EDV, ESV, and EF, the clinician could then consider opting for CMR for higher resolution images for better assessments of the trabecular mass and these key prognostic indicators.

We show, in accordance with previous studies on normal and hypertrophic hearts (Jaspers et al., 2013; Zhan et al., 2024), that contouring of the trabeculations impacts on volume measurements. Because we have based our investigations on individuals with excessive trabeculation, the effects of contouring are pronounced. In our population, our best contouring led to a halving of the population with an EF less than 40%, and it doubled the number of individuals with an EF greater than 50%. In effect, many of our current manners of assessing LV function bias toward reduced function when there is a setting of excessive trabeculation. More accurate volume measurements may also have important clinical implications in patients with excessive trabeculation and heart failure (HF). Thus, half of the patients with an EF of less than 40% would no longer have an indication to initiate some of the four treatment pillars in HF, according to the current guidelines (McDonagh et al., 2021). There could be a significant reduction in indications for device therapies. Moreover, a higher EF than previously reported would explain the overall good prognosis in individuals with excessive trabeculation over 9.5 years follow‐up (Zemrak et al., 2014).

**FIGURE 7:**
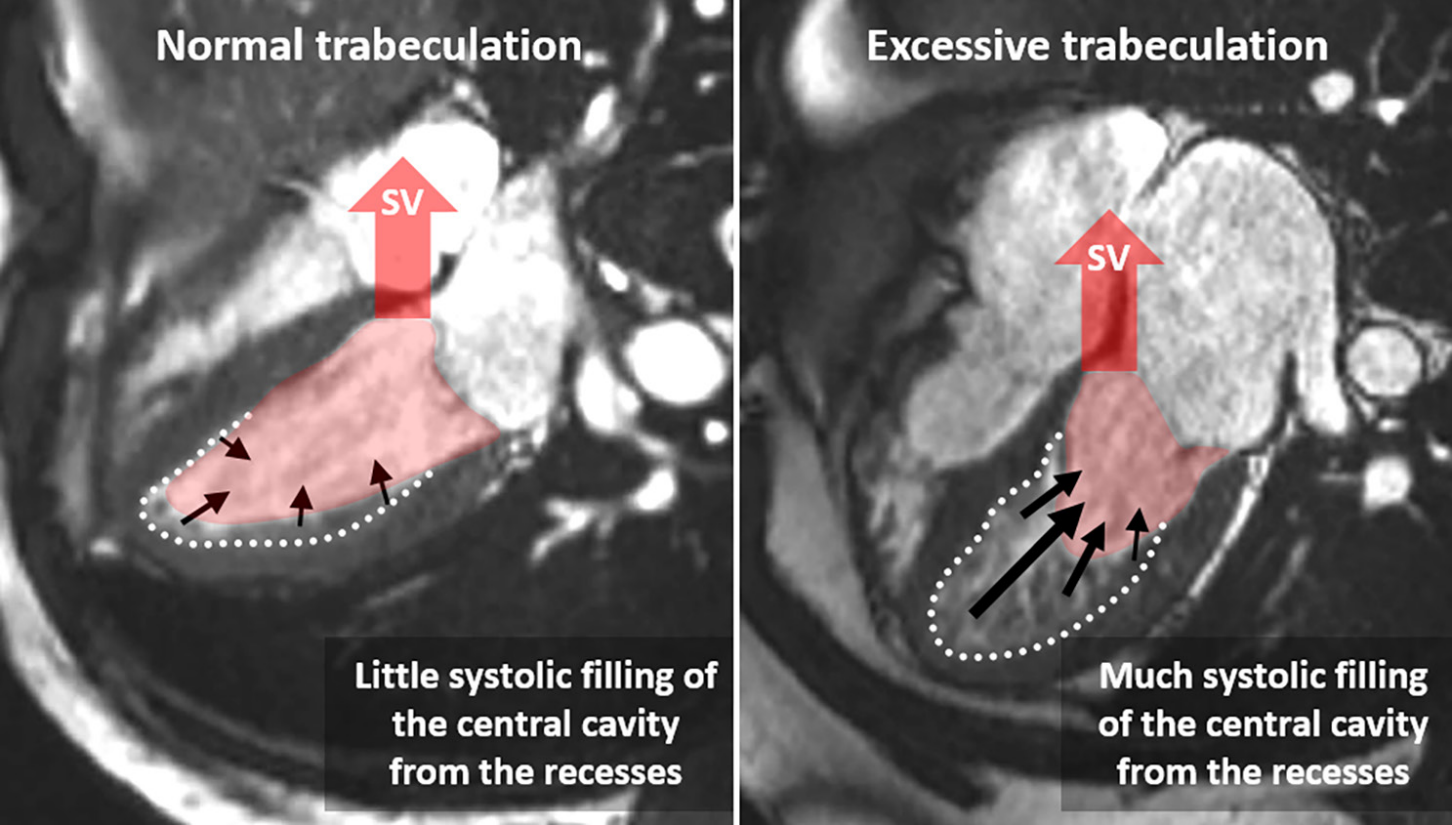
Systolic filling of the central cavity (CC) from the IR. In the normal left ventricle (LV) (left‐hand image), the IR are only a small fraction of the total LV cavity and their ejection has a small effect on the systolic volume of the CC. In the excessively trabeculated LV (right‐hand image), the IR are a large fraction of the total LV cavity and their ejection has a substantial inflating effect on the systolic volume of the CC. If only the CC is analyzed, the setting of excessive trabeculation can appear to be in a state of severely reduced function, even if the actual ejection fraction and stroke volume is comparable to that of the normal LV.

The trabecular layer, however, cannot be analyzed in isolation. Like the ventricular cavity, it is ultimately enclosed by the compact wall. We presume that the high EF of the IR reflects the work done both by the trabecular layer and the compact wall. In addition, the high EF of the trabecular layer likely reflects the IR eject into the CC, whereas the systolic CC is a conduit (Figure 7). In systole, the CC will empty into the aorta while concurrently it will be filled with blood from the IR (Figure 7). Therefore, the greater the trabecular layer, and the greater the systolic compression of the IR, the greater the systolic filling will be of the CC. In this way, a greater trabecular layer biases toward a lower measured EF of the CC, even if the SV is in the normal range relative to the LV mass and volume. Interestingly, the trabecular layer may intrinsically allow for a greater EF than the compact wall, given that in the individuals with a total EF above 50%, their IR operated with an average EF of 74%. Indeed, animals with highly trabeculated ventricles can achieve EF of approximately 90% (Williams et al., 2019).

### 4.1 Study limitations

The fractional area change and volume readouts reported here derive from manual labelling of the structures. Some challenges of this approach (Luu et al., 2022) are overcome by the use of a threshold value to separate cavity from myocardium. Such threshold can be determined on a case‐by‐case basis as there are slight variations between individual recording sessions. We applied the same threshold in diastole and systole as it seemed the simplest approach to us, but this approach may not be the best. The use of thresholds is inadequate to separate compact from trabecular myocardium, and to separate the CC from IR. For these distinctions, one relies on identification of landmarks, such as the depth of IR, and “common sense” assumptions, such as that the compact wall will take the shape of a ring of more or less even thickness. This manner of analysis, then, necessarily comes with some technical variation. The difference in EF of the IR versus the CC, however, is so great that this finding is very resilient to variation labeling of structures, as we show in Data S1. Being a CMR imaging‐focused study, we did not include other clinical data. Only a small sample size, from a single center, was included in this proof of concept study. We selected only patients with a positive Jacquier criterion, in order to analyze a well‐defined trabecular layer.

## 5 CONCLUSIONS

The trabecular layer is associated with a high EF. By contouring the trabeculations, LV volumes are more accurately assessed, and implicitly the EF. Stroke volume, in contrast, is not affected as long as the trabecular mass is measured similarly in diastole and systole. The contour‐EF, which actually is much higher, could better guide the clinical management in daily practice, avoiding inappropriate diagnosis and treatment.

## AUTHOR CONTRIBUTIONS

B.J. and D.V. conceived and designed research; I.S.V., R.C.R., A.I.N., and B.J. analyzed data; I.S.V., R.C.R., A.I.N., D.V., and B.J interpreted results of experiments; B.J. and I.S.V. prepared figures; B.J. and I.S.V. drafted manuscript; I.S.V., R.C.R., A.I.N., D.V., and B.J. edited and revised manuscript; I.S.V., R.C.R., A.I.N., D.V., and B.J. approved final version of manuscript.

## FUNDING INFORMATION

This study was partly funded by a National Grant of the Romanian Ministry of Research and Innovation: Heart Preserved/project No. PN‐III‐P1‐1.1‐TE‐2016‐0669 (to R.C.R., A.I.N., and D.V.).

## CONFLICT OF INTEREST STATEMENT

The authors have no conflicts of interest to disclose.

## ETHICS STATEMENT

The study was conducted in accordance with the Declaration of Helsinki, and approved by the Research Ethics Committee of the University and Emergency Hospital, Bucharest, Romania (no: 74155). All participants provided informed written consent.

## Supporting information


Data S1.

## Data Availability

Data will be made available upon reasonable request.

## References

[phy216101-bib-0001] Barskiy, V. (2023a). Arrhythmogenic right ventricular cardiomyopathy. Case Study. 10.53347/rID-69431

[phy216101-bib-0002] Barskiy, V. (2023b). Non‐compaction of the left ventricle. Case Study. 10.53347/rID-69436

[phy216101-bib-0003] Chan, V. S. , Chan, C. W. , & Cheung, S. C. (2019). Cardiac magnetic resonance imaging in the diagnosis of biventricular non‐compaction in a young but failing heart. Hong Kong Medical Journal, 25, 330.e1–330.e2. 10.12809/hkmj187709 31416995

[phy216101-bib-0004] Choi, Y. , Kim, S. M. , Lee, S.‐C. , Chang, S.‐A. , Jang, S. Y. , & Choe, Y. H. (2016). Quantification of left ventricular trabeculae using cardiovascular magnetic resonance for the diagnosis of left ventricular non‐compaction: Evaluation of trabecular volume and refined semi‐quantitative criteria. Journal of Cardiovascular Magnetic Resonance, 18, 24. 10.1186/s12968-016-0245-2 27142637 PMC4855408

[phy216101-bib-0005] Ciezarek, A. , Gardner, L. , Savolainen, V. , & Block, B. (2020). Skeletal muscle and cardiac transcriptomics of a regionally endothermic fish, the Pacific bluefin tuna, *Thunnus orientalis* . BMC Genomics, 21, 642. 10.1186/s12864-020-07058-z 32942994 PMC7499911

[phy216101-bib-0006] Davies, R. H. , Augusto, J. B. , Bhuva, A. , Xue, H. , Treibel, T. A. , Ye, Y. , Hughes, R. K. , Bai, W. , Lau, C. , Shiwani, H. , Fontana, M. , Kozor, R. , Herrey, A. , Lopes, L. R. , Maestrini, V. , Rosmini, S. , Petersen, S. E. , Kellman, P. , Rueckert, D. , … Moon, J. C. (2022). Precision measurement of cardiac structure and function in cardiovascular magnetic resonance using machine learning. Journal of Cardiovascular Magnetic Resonance, 24, 16. 10.1186/s12968-022-00846-4 35272664 PMC8908603

[phy216101-bib-0007] de la Chica, J. A. , Gómez‐Talavera, S. , García‐Ruiz, J. M. , García‐Lunar, I. , Oliva, B. , Fernández‐Alvira, J. M. , López‐Melgar, B. , Sánchez‐González, J. , de la Pompa, J. L. , Mendiguren, J. M. , Martínez de Vega, V. , Fernández‐Ortiz, A. , Sanz, J. , Fernández‐Friera, L. , Ibáñez, B. , & Fuster, V. (2020). Association between left ventricular noncompaction and vigorous physical activity. Journal of the American College of Cardiology, 76, 1723–1733. 10.1016/j.jacc.2020.08.030 33032733

[phy216101-bib-0008] Dewey, M. , Siebes, M. , Kachelrieß, M. , Kofoed, K. F. , Maurovich‐Horvat, P. , Nikolaou, K. , Bai, W. , Kofler, A. , Manka, R. , Kozerke, S. , Chiribiri, A. , Schaeffter, T. , Michallek, F. , Bengel, F. , Nekolla, S. , Knaapen, P. , Lubberink, M. , Senior, R. , Tang, M.‐X. , … Schreiber, L. (2020). Clinical quantitative cardiac imaging for the assessment of myocardial ischaemia. Nature Reviews. Cardiology, 17, 427–450. 10.1038/s41569-020-0341-8 32094693 PMC7297668

[phy216101-bib-0009] Dreisbach, J. G. , Mathur, S. , Houbois, C. P. , Oechslin, E. , Ross, H. , Hanneman, K. , & Wintersperger, B. J. (2020). Cardiovascular magnetic resonance based diagnosis of left ventricular non‐compaction cardiomyopathy: Impact of cine bSSFP strain analysis. Journal of Cardiovascular Magnetic Resonance, 22, 9. 10.1186/s12968-020-0599-3 31996239 PMC6990516

[phy216101-bib-0010] Faber, J. W. , Wüst, R. C. I. , Dierx, I. , Hummelink, J. A. , Kuster, D. W. D. , Nollet, E. , Moorman, A. F. M. , Sánchez‐Quintana, D. , van der Wal, A. C. , Christoffels, V. M. , & Jensen, B. (2022). Equal force generation potential of trabecular and compact wall ventricular cardiomyocytes. iScience, 25, 105393. 10.1016/j.isci.2022.105393 36345331 PMC9636041

[phy216101-bib-0011] Grothoff, M. , Pachowsky, M. , Hoffmann, J. , Posch, M. , Klaassen, S. , Lehmkuhl, L. , & Gutberlet, M. (2012). Value of cardiovascular MR in diagnosing left ventricular non‐compaction cardiomyopathy and in discriminating between other cardiomyopathies. European Radiology, 22, 2699–2709. 10.1007/s00330-012-2554-7 22772366 PMC3486997

[phy216101-bib-0012] Jacquier, A. , Thuny, F. , Jop, B. , Giorgi, R. , Cohen, F. , Gaubert, J. Y. , Vidal, V. , Bartoli, J. M. , Habib, G. , & Moulin, G. (2010). Measurement of trabeculated left ventricular mass using cardiac magnetic resonance imaging in the diagnosis of left ventricular non‐compaction. European Heart Journal, 31, 1098–1104. 10.1093/eurheartj/ehp595 20089517

[phy216101-bib-0013] Jaspers, K. , Freling, H. G. , Van Wijk, K. , Romijn, E. I. , Greuter, M. J. W. , & Willems, T. P. (2013). Improving the reproducibility of MR‐derived left ventricular volume and function measurements with a semi‐automatic threshold‐based segmentation algorithm. The International Journal of Cardiovascular Imaging, 29, 617–623. 10.1007/s10554-012-0130-5 23053857

[phy216101-bib-0014] Kaplan‐List, K. (2023). Secundum atrial septal defect. Case study. 10.53347/rID-42823

[phy216101-bib-0015] Keshavamurthy, J. (2023). Incidental infundibular pulmonary stenosis and left sided superior vena cava. Case Study. 10.53347/rID-44210

[phy216101-bib-0016] Lang, R. M. , Badano, L. P. , Victor, M. A. , Afilalo, J. , Armstrong, A. , Ernande, L. , Flachskampf, F. A. , Foster, E. , Goldstein, S. A. , Kuznetsova, T. , Lancellotti, P. , Muraru, D. , Picard, M. H. , Retzschel, E. R. , Rudski, L. , Spencer, K. T. , Tsang, W. , & Voigt, J. U. (2015). Recommendations for cardiac chamber quantification by echocardiography in adults: An update from the American Society of Echocardiography and the European Association of Cardiovascular Imaging. Journal of the American Society of Echocardiography, 28, 1–39.e14. 10.1016/j.echo.2014.10.003 25559473

[phy216101-bib-0017] Litviňuková, M. , Talavera‐López, C. , Maatz, H. , Reichart, D. , Worth, C. L. , Lindberg, E. L. , Kanda, M. , Polanski, K. , Heinig, M. , Lee, M. , Nadelmann, E. R. , Roberts, K. , Tuck, L. , Fasouli, E. S. , DeLaughter, D. M. , McDonough, B. , Wakimoto, H. , Gorham, J. M. , Samari, S. , … Teichmann, S. A. (2020). Cells of the adult human heart. Nature, 588, 466–472. 10.1038/s41586-020-2797-4 32971526 PMC7681775

[phy216101-bib-0018] Luijkx, T. (2023a). Left ventricular non‐compaction. Case Study. 10.53347/rID-39933

[phy216101-bib-0019] Luijkx, T. (2023b). Physiological cardiac adaptation to exercise. Case Study. 10.53347/rID-39934

[phy216101-bib-0020] Luu, J. M. , Gebhard, C. , Ramasundarahettige, C. , Desai, D. , Schulze, K. , Marcotte, F. , Awadalla, P. , Broet, P. , Dummer, T. , Hicks, J. , Larose, E. , Moody, A. , Smith, E. E. , Tardif, J.‐C. , Teixeira, T. , Teo, K. K. , Vena, J. , Lee, D. S. , Anand, S. S. , & Friedrich, M. G. (2022). Normal sex and age‐specific parameters in a multi‐ethnic population: A cardiovascular magnetic resonance study of the Canadian Alliance for healthy hearts and minds cohort. Journal of Cardiovascular Magnetic Resonance, 24, 2. 10.1186/s12968-021-00819-z 34980185 PMC8722350

[phy216101-bib-0021] Martins, E. , Pinho, T. , Carpenter, S. , Leite, S. , Garcia, R. , Madureira, A. , & Oliveira, J. P. (2014). Histopathological evidence of Fabry disease in a female patient with left ventricular noncompaction. Revista Portuguesa de Cardiologia, 33(565), e1–565.e6. 10.1016/j.repc.2014.02.021 25246064

[phy216101-bib-0022] McDonagh, T. A. , Metra, M. , Adamo, M. , Gardner, R. S. , Baumbach, A. , Böhm, M. , Burri, H. , Butler, J. , Čelutkienė, J. , Chioncel, O. , Cleland, J. G. F. , Coats, A. J. S. , Crespo‐Leiro, M. G. , Farmakis, D. , Gilard, M. , Heymans, S. , Hoes, A. W. , Jaarsma, T. , Jankowska, E. A. , … Skibelund, A. K. (2021). 2021 ESC guidelines for the diagnosis and treatment of acute and chronic heart failure. European Heart Journal, 42, 3599–3726. 10.1093/eurheartj/ehab368 34447992

[phy216101-bib-0023] Meyer, H. V. , Dawes, T. J. W. , Serrani, M. , Bai, W. , Tokarczuk, P. , Cai, J. , de Marvao, A. , Henry, A. , Lumbers, R. T. , Gierten, J. , Thumberger, T. , Wittbrodt, J. , Ware, J. S. , Rueckert, D. , Matthews, P. M. , Prasad, S. K. , Costantino, M. L. , Cook, S. A. , Birney, E. , & O'Regan, D. P. (2020). Genetic and functional insights into the fractal structure of the heart. Nature, 584, 589–594. 10.1038/s41586-020-2635-8 32814899 PMC7116759

[phy216101-bib-0024] O'Rourke, R. (2023). Endocardial fibroelastosis. Case Study. 10.53347/rID-86346

[phy216101-bib-0025] Paluszkiewicz, J. , Milting, H. , Kałużna‐Oleksy, M. , Pyda, M. , Janus, M. , Körperich, H. , & Piran, M. (2022). Left ventricular non‐compaction cardiomyopathy‐still more questions than answers. Journal of Clinical Medicine, 11, 4135. 10.3390/jcm11144135 35887898 PMC9315982

[phy216101-bib-0026] Petersen, S. E. , Jensen, B. , Aung, N. , Friedrich, M. G. , McMahon, C. J. , Mohiddin, S. A. , Pignatelli, R. H. , Ricci, F. , Anderson, R. H. , & Bluemke, D. A. (2023). Excessive trabeculation of the left ventricle: JACC: Cardiovascular imaging expert panel paper. JACC: Cardiovascular Imaging, 16, 408–425. 10.1016/j.jcmg.2022.12.026 36764891 PMC9988693

[phy216101-bib-0027] Polacin, M. , Károlyi, M. , Wilzeck, V. , Eberhard, M. , Gotschy, A. , Alkadhi, H. , Kozerke, S. , & Manka, R. (2022). Three‐dimensional whole‐heart cardiac MRI sequence for measuring trabeculation in left ventricular noncompaction. Radiology: Cardiothoracic Imaging, 4(6), e220109. 10.1148/ryct.220109 36601458 PMC9806726

[phy216101-bib-0028] Positano, V. , Meloni, A. , Macaione, F. , Santarelli, M. F. , Pistoia, L. , Barison, A. , Novo, S. , & Pepe, A. (2018). Non‐compact myocardium assessment by cardiac magnetic resonance: Dependence on image analysis method. The International Journal of Cardiovascular Imaging, 34, 1227–1238. 10.1007/s10554-018-1331-3 29524076

[phy216101-bib-0045] Rangarajan, V. , Chacko, S. J. , Romano, S. , Jue, J. , Jariwala, N. , Chung, J. , & Farzaneh‐Far, A. (2016). Left ventricular long axis function assessed during cine‐cardiovascular magnetic resonance is an independent predictor of adverse cardiac events. Journal of Cardiovascular Magnetic Resonance, 18, 1–10. 10.1186/s12968-016-0257-y 27266262 PMC4897936

[phy216101-bib-0029] Reyes, J. A. , Dipchand, A. I. , & Chiasson, D. A. (2022). Paediatric dilated cardiomyopathy with and without endocardial fibroelastosis—A pathological analysis of 89 explants. Cardiology in the Young, 32, 1041–1047. 10.1017/S1047951121003590 34486505

[phy216101-bib-0030] Riekerk HCE , Coolen BF , J. Strijkers G , van der Wal AC , Petersen SE , Sheppard MN , Oostra RJ , Christoffels VM , Jensen B. Higher spatial resolution improves the interpretation of the extent of ventricular trabeculation. Journal of Anatomy 240: 357–375, 2022. 10.1111/joa.13559. 34569075 PMC8742974

[phy216101-bib-0031] Riffel, J. H. , Andre, F. , Maertens, M. , Rost, F. , Keller, M. G. P. , Giusca, S. , Seitz, S. , Kristen, A. V. , Müller, M. , Giannitsis, E. , Korosoglou, G. , Katus, H. A. , & Buss, S. J. (2015). Fast assessment of long axis strain with standard cardiovascular magnetic resonance: A validation study of a novel parameter with reference values. Journal of Cardiovascular Magnetic Resonance, 17, 1–9. 10.1186/s12968-015-0171-8 26253220 PMC4529700

[phy216101-bib-0032] Ross, S. B. , Jones, K. , Blanch, B. , Puranik, R. , McGeechan, K. , Barratt, A. , & Semsarian, C. (2020). A systematic review and meta‐analysis of the prevalence of left ventricular non‐compaction in adults. European Heart Journal, 41, 1428–1436b. 10.1093/eurheartj/ehz317 31143950

[phy216101-bib-0033] Sheehy, N. (2023). Apical hypertrophic cardiomyopathy. Case Study. 10.53347/rID-79346

[phy216101-bib-0034] Sigvardsen, P. E. , Fuchs, A. , Kühl, J. T. , Afzal, S. , Køber, L. , Nordestgaard, B. G. , & Kofoed, K. F. (2021). Left ventricular trabeculation and major adverse cardiovascular events: The Copenhagen general population study. European Heart Journal Cardiovascular Imaging, 22, 67–74. 10.1093/ehjci/jeaa110 32386205

[phy216101-bib-0035] Stacey, R. B. , Andersen, M. M. , St. Clair, M. , Hundley, W. G. , & Thohan, V. (2013). Comparison of systolic and diastolic criteria for isolated LV noncompaction in CMR. JACC: Cardiovascular Imaging, 6, 931–940. 10.1016/j.jcmg.2013.01.014 23769489

[phy216101-bib-0036] Takamatsu, M. , Kamohara, K. , Sato, M. , & Koga, Y. (2020). Effect of noncompacted myocardial resection on isolated left ventricular noncompaction. The Annals of Thoracic Surgery, 110, e387–e389. 10.1016/j.athoracsur.2020.03.071 32360189

[phy216101-bib-0037] Thut, T. , Valsangiacomo Büchel, E. , Geiger, J. , Kellenberger, C. J. , Rücker, B. , & Burkhardt, B. E. U. (2023). Signal thresholding segmentation of ventricular volumes in young patients with various diseases—Can we trust the numbers? Diagnostics, 13, 180. 10.3390/diagnostics13020180 36672990 PMC9857934

[phy216101-bib-0038] Tigges, S. (2023). Left ventricular non‐compaction. Case Study. 10.53347/rID-158103

[phy216101-bib-0039] Williams, C. J. A. , Greunz, E. M. , Ringgaard, S. , Hansen, K. , Bertelsen, M. F. , & Wang, T. (2019). Magnetic resonance imaging (MRI) reveals high cardiac ejection fractions in red‐footed tortoises (*Chelonoidis carbonarius*). Journal of Experimental Biology, 222(18), jeb206714. 31439654 10.1242/jeb.206714

[phy216101-bib-0040] Woodbridge, S. P. , Aung, N. , Paiva, J. M. , Sanghvi, M. M. , Zemrak, F. , Fung, K. , & Petersen, S. E. (2019). Physical activity and left ventricular trabeculation in the UK Biobank community‐based cohort study. Heart, 105, 990–998. 10.1136/heartjnl-2018-314155 30723101 PMC6582810

[phy216101-bib-0041] Yarmola, I. (2023). Dilated cardiomyopathy with non‐compaction of the left ventricle. Case Study. 10.53347/rID-69559

[phy216101-bib-0042] Yousef, Z. R. , Foley, P. W. X. , Khadjooi, K. , Chalil, S. , Sandman, H. , Mohammed, N. U. H. , & Leyva, F. (2009). Left ventricular non‐compaction: Clinical features and cardiovascular magnetic resonance imaging. BMC Cardiovascular Disorders, 9, 37. 10.1186/1471-2261-9-37 19664240 PMC2743643

[phy216101-bib-0043] Zemrak, F. , Ahlman, M. A. , Captur, G. , Mohiddin, S. A. , Kawel‐Boehm, N. , Prince, M. R. , Moon, J. C. , Hundley, W. G. , Lima, J. A. C. , Bluemke, D. A. , & Petersen, S. E. (2014). The relationship of left ventricular trabeculation to ventricular function and structure over a 9.5‐year follow‐up. Journal of the American College of Cardiology, 64, 1971–1980. 10.1016/j.jacc.2014.08.035 25440091 PMC4610345

[phy216101-bib-0044] Zhan, Y. , Friedrich, M. G. , Dendukuri, N. , Lu, Y. , Chetrit, M. , Schiller, I. , Joseph, L. , Shaw, J. L. , Chuang, M. L. , Riffel, J. H. , Manning, W. J. , & Afilalo, J. (2024). Meta‐analysis of normal reference values for right and left ventricular quantification by cardiovascular magnetic resonance. Circulation. Cardiovascular Imaging, 17, e016090. 10.1161/CIRCIMAGING.123.016090 38377242

