# Supplementary material for: High ejection fraction of the left ventricular trabecular layer of the human heart
Source: Physiol Rep. 2024 Jun 21;12(12):e16101. doi: 10.14814/phy2.16101 (PMC11192606; doi:10.14814/phy2.16101)
Supplement: Supplementary file 1 — Data S1. [file PHY2-12-e16101-s001.docx]

**SUPPLEMENTAL MATERIAL**

**High ejection fraction of the left ventricular trabecular layer of the human heart**

**Running title: Trabecular layer ejection fraction**

Ionela-Simona Visoiu^1^, Roxana Cristina Rimbas^1,2^, Alina Ioana Nicula^1,3^, Dragos Vinereanu^1,2^, Bjarke Jensen^4^

^1^Department of Cardiology and Cardiovascular Surgery, University of Medicine and Pharmacy Carol Davila, Bucharest, Romania

^2^Department of Cardiology, University and Emergency Hospital, Bucharest, Romania

^3^Department of Radiology, University and Emergency Hospital, Bucharest, Romania

^4^Department of Medical Biology, Amsterdam Cardiovascular Sciences, University of Amsterdam, Amsterdam UMC, Amsterdam, the Netherlands

**Corespondence:** Bjarke Jensen ([b.jensen@amsterdamumc.nl](mailto:b.jensen@amsterdamumc.nl))


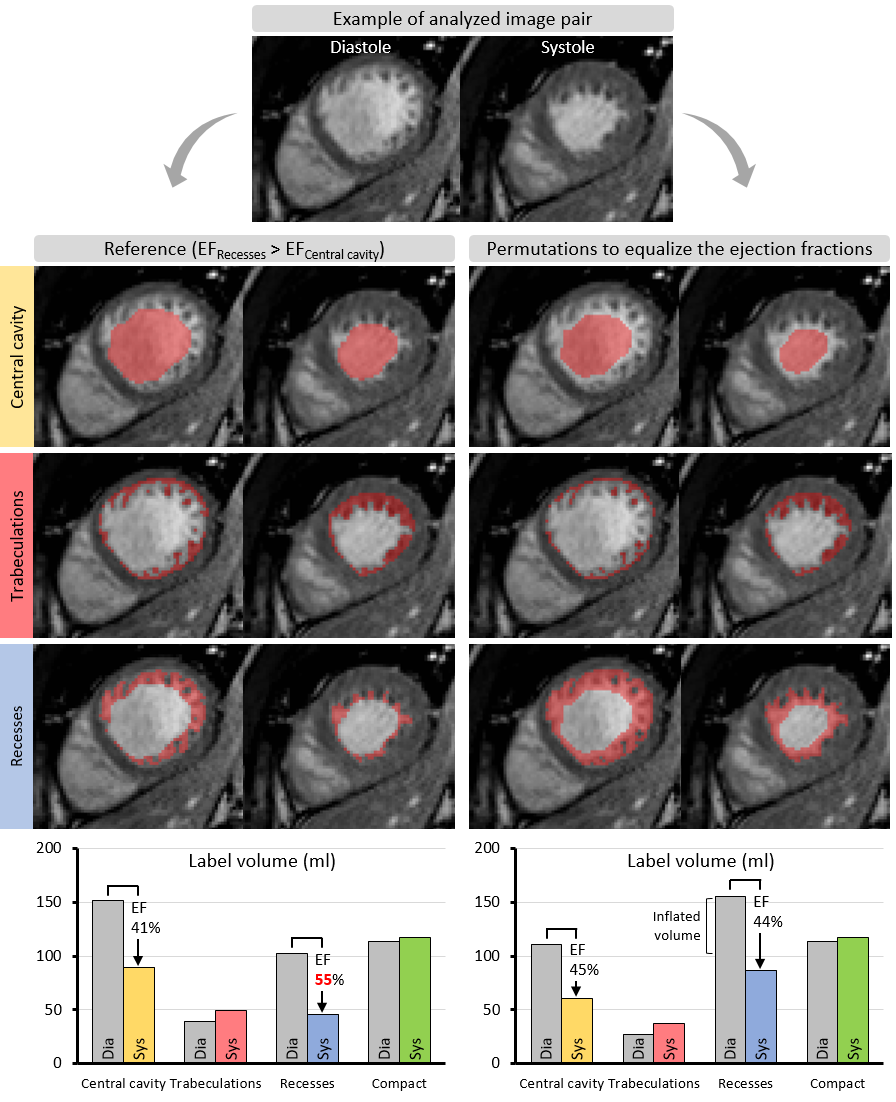


**Supplemental Figure S1**. Very substantial mislabeling of the intertrabecular recesses (inflation) is required, at the cost of the central cavity and trabeculations labels, to lower the EF of the intertrabecular recesses to the EF of the central cavity (in this case from 55 to 44%).


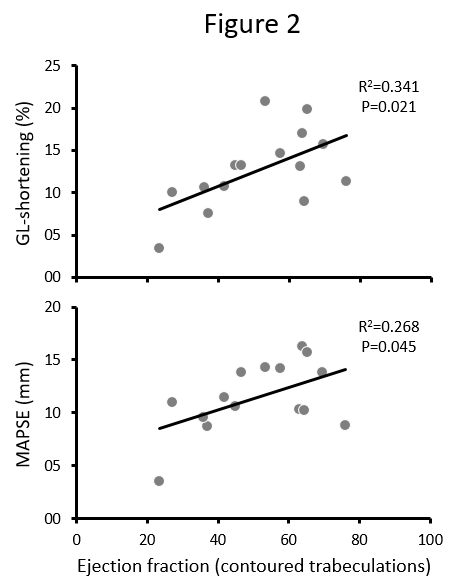


**Supplemental Figure S2**. The ejection fraction derived from frames with contoured trabeculations was significantly correlated to both global longitudinal shortening (GL-Shortening) and mitral annular plane systolic excursion (MAPSE). P-values relate to linear regressions.


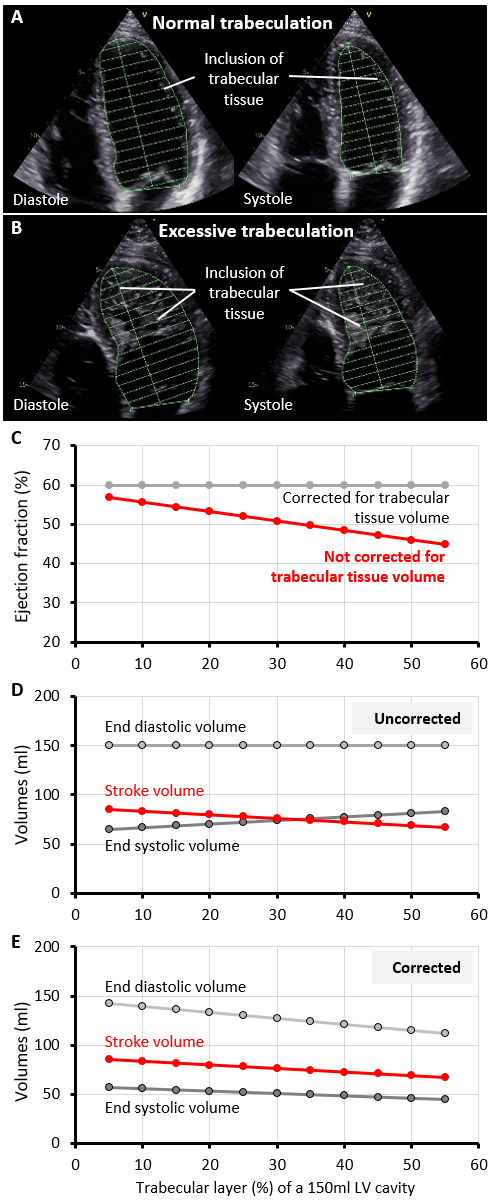


**Supplemental Figure S3.** Impact of including trabeculations in the LV blood pool. **A**-**B**. Echocardiography of a left ventricle with a normal extent of trabeculations (**A**) and with excessive trabeculations (**B**). **C**. The greater the proportion of the trabecular layer, the greater the reduction in ejection fraction. **D**. More trabeculations equates more un-ejectable tissue in the cavity as define per guidelines (Lang et al), which increases the end systolic volume and thus diminishes the stroke volume. **E**. If the un-ejectable trabeculations are corrected for by accurate contouring, all volumes diminish isometrically.


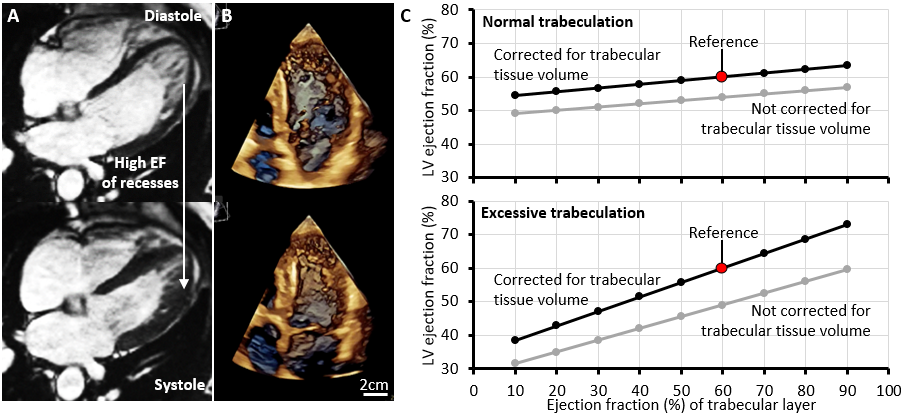


**Supplemental Figure S4.** Impact of trabecular layer ejection fractions on total ejection fraction. **A**. CMR of a case of excessive trabeculation with a high ejection fraction of the trabecular layer. **B**. Same case as in A, visualized with 3D rendering of echocardiography, showing substantial compression of the intertrabecular recesses. **C**. The greater the ejection fraction of the trabecular layer, the greater the ejection of the total ventricular cavity (while the central cavity ejection fraction is kept at 60). Although this effect is subtle in a setting of normal trabeculation, the effect can be substantial in a setting of excessive trabeculation.


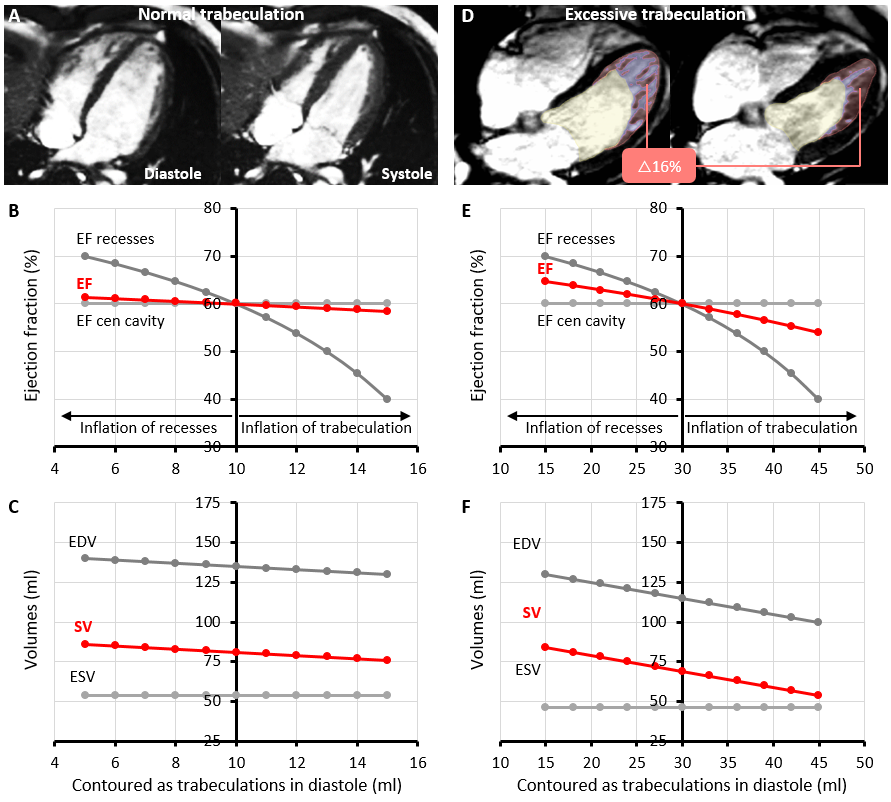


**Supplemental Figure S5.** Theoretical calculations on the effect of discrepancies in trabecular myocardium measurements between diastole and systole. **A**-**C**. In a setting of normal trabeculation, in systole the trabeculations may be measured to comprise 10ml. Given this proportionally small volume, even substantially different labelling of the trabeculations in diastole, does not affect much the measured EF. **D**-**F**. In a setting of excessive trabeculation, in systole the trabeculations may be measured to comprise 30ml. Substantially different labelling of the trabeculations in diastole affects EDV, SV, and EF. EDV, end diastolic volume; ESV, end systolic volume; SV stroke volume; EF, ejection fraction.
